# The optimal introversion angle and length of pedicle screw to avoid L1-S1 vascular damage

**DOI:** 10.1186/s12893-024-02483-3

**Published:** 2024-06-21

**Authors:** Ying Chen, Junyi Yang, Jie Liang, Weifei Wu

**Affiliations:** 1 Department of Orthopedics, The First College of Clinical Medical Science, China Three Gorges University, Hubei, China; 2 Yichang Central People’s Hospital Hubei, Hubei, China

**Keywords:** Pedicle screw placement, Optimal INTA and depth, Lumbosacral vessel injury

## Abstract

**Background:**

posterior pedicle screw fixation is common method, one of the most severe complications is iatrogenic vascular damage, no report investigated association of different introversion angles (INTAs) and length of pedicle screw. The aims were to investigate the optimal introversion angle and length of pedicle screw for improving the safety of the operation, and to analyze the differences of vascular damage types at L1-S1.

**Methods:**

Lumbar CT imaging data from110 patients were analyzed by DICOM software, and all parameters were measured by new Cartesian coordinate system, INTAs (L1-L5:5°,10°,15°,S1: 0°, 5°,10°,15°), D_O−AVC_ (the distance between the origin (O) with anterior vertebral cortex (AVC)), D_AVC−PGVs_ (the distance between AVC and the prevertebral great vessels (PGVs)), D_O−PGVs_ (the distance between the O and PGVs). At different INTAs, D_AVC−PGVs_ were divided into four grades: Grade III: D_AVC−PGVs_ ≤ 3 mm, Grade II: 3 mm < D_AVC−PGVs_ ≤ 5 mm, Grade I: D_AVC−PGVs_ > 5 mm, and N: the not touching PGVs.

**Results:**

The optimal INTA was 5° at L1-L3, the left was 5° and the right was 15° at L4, and screw length was less than 50 mm at L1-L4. At L5, the left optimal INTA was 5° and the right was 10°, and screw length was less than 45 mm. The optimal INTA was 15° at S1, and screw length was less than 50 mm. However, screw length was less than 40 mm when the INTA was 0° or 5° at S1.

**Conclusions:**

At L5-S1, the risk of vascular injury is the highest. INTA and length of the pedicle screw in lumbar operation are closely related. 3 mm interval of screw length may be more preferable to reduce vascular damage.

## Introduction

Posterior lumbosacral fixation with pedicle screw is common method for treatment various spinal diseases. However, one of the most severe complication of spinal surgery is iatrogenic vascular damage [1–4]. In spite of the low incidence (less than 1%) of vascular injury during posterior spine surgery, the mortality rate is as high as 61% if the iliac artery and aorta are injured [3, 5]. Computer assist system can improve pedicle screw accuracy, but can’t completely avoid vessel damage [6, 7]. Hence, mastering the optimal introversion angles (INTAs) and insertion depth of pedicle screws can improve the safety of operation. To our knowledge, although a few studies focused on the relationship between INTA and prevertebral great vessels (PGVs) [8, 9], no study investigated association of different INTAs and length of pedicle screw. Therefore, the aims of this study were to investigate the maximum safe distance between pedicle screw entry point and the edge of the blood vessel, and to analyze the relationship of pedicle screw long contacting with PGVs and different INTAs.

## Materials and methods

### Subjects

Patients of orthopedic inpatient department with lumbar disc herniation (LDH) from January 1, 2019 to December 31, 2021 were included. The inclusion criteria were: age 40 to 80 years, intact lumbosacral CT data from L1-S1.Exclusion criteria: (1) imaging data with unclear. (2) Patients diagnosed with lumbar deformity, lumbar fractures, tumors tuberculosis, spondylolisthesis. (3) Patients with history of retroperitoneal surgery or spinal surgery. Before the study was carried out, the Ethics Committee of the People’s Hospital of China Three Gorges University had approved the research plan. All participants signed an informed consent allowing their clinical data to be used for the research study.

### CT image measurement

**Fig. 1:**
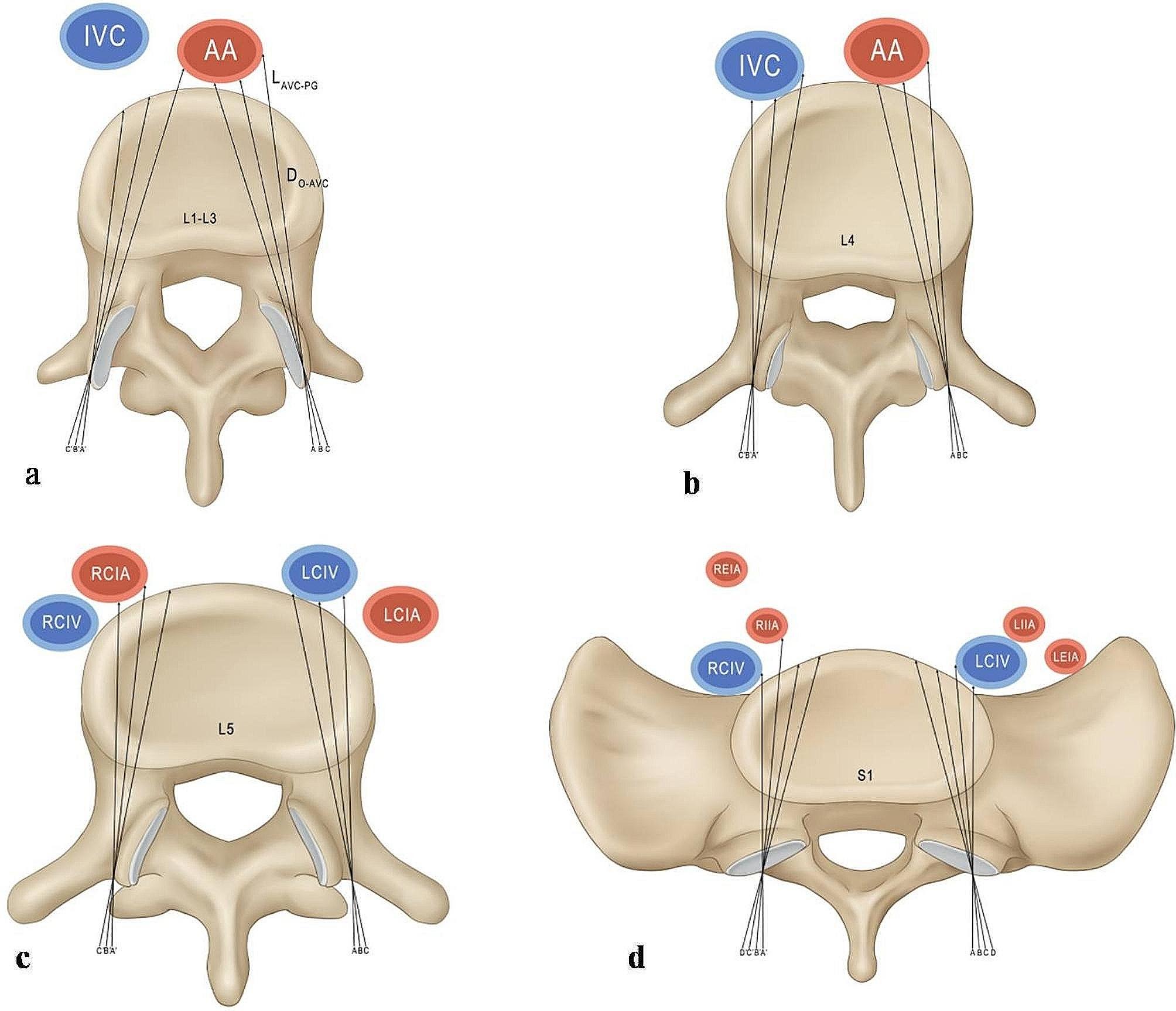
L_AVC−PGV_ and D_O−AVC_ with different INTAs in L1-S1, S_O−AVC_= L_AVC−PGV_+ D_O−AVC_. AA: abdominal aorta, IVC: inferior vena cava, LCIA: left common iliac artery, RCIA: right common iliac artery, LCIV: left common iliac vein, RCIV: right common iliac vein. LEIA: left external iliac artery, LIIA: left internal iliac artery, REIA: right external iliac artery, RIIA: right internal iliac artery, D_AVC−PGV_: the distance between AVC and PGV, AVC: anterior vertebral cortex, PGV: prevertebral great vessels, INTA: introversion angles, L: lumbar, S: sacral. At L1-L5 (figure **a**, **b**, **c**), A, B and C represent the left INTA with 5°, 10° and 15°, respectively; A′, B′ and C′ represent the right INTA with 5°, 10° and 15° respectively. At S1 (figure **d**), A, B, C ,D represent the left INTA at 0°, 5°, 10° and 15°, respectively; A′, B′, C′ and D’ represent the right INTA at 0°, 5°, 10° and 15° respectively

**Fig. 2:**
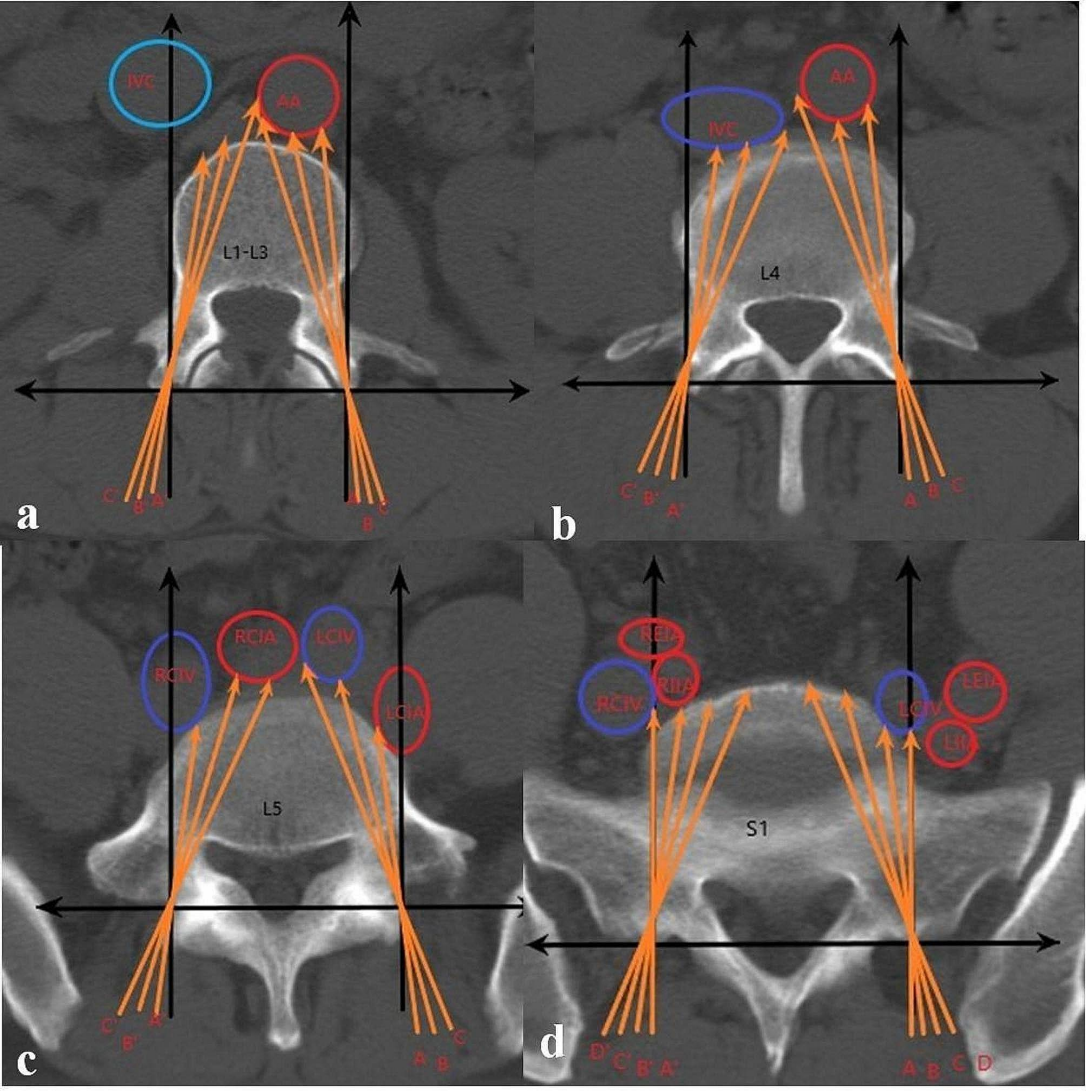
The distance from the needle insertion point to the adjacent blood vessels of the vertebral body from CT images. AA: abdominal aorta, IVC: inferior vena cava, LCIA: left common iliac artery, RCIA: right common iliac artery, LCIV: left common iliac vein, RCIV: right common iliac vein. LEIA: left external iliac artery, LIIA: left internal iliac artery, REIA: right external iliac artery, RIIA: right internal iliac artery, INTA: introversion angles, L: lumbar, S: sacral. At L1-L5 (figure **a**, **b**, **c**), A, B and C represent the left INTA with 5°, 10° and 15°, respectively; A′, B′ and C′ represent the right INTA with 5°, 10° and 15° respectively. At S1 (figure **d**), A, B, C ,D represent the left INTA at 0°, 5°, 10° and 15°, respectively; A′, B′, C′ and D’ represent the right INTA at 0°, 5°, 10° and 15° respectively

L1-S1 was scanned by dual-source spiral CT, and then images were imported to PACS workstation. All images were analyzed by DICOM software. The image (the cross-section of each lumbosacral vertebral body) passing through the widest plane of the bilateral pedicles was selected as all parameters’ measurement. The new Cartesian coordinate system developed by Takeshita et al. was used to construct the trajectory of pedicle screw [10], and the following parameters were measured (Figs. 1 and 2):

(1) New Cartesian coordinate system: the origin point (O) was defined as the middle of the base of the superior facet, which was the entry point of the pedicle screw. A line connecting the middle points of both bases of the superior facets was defined as the X-axis, the line passing through the O and perpendicular to the X-axis was defined as the Y-axis.

(2) INTA: the angle between the pedicle screw trajectory with Y axis (L1-L5: 5°, 10°, 15°. S1: 0°, 5°, 10°, 15°), it was assumed that pedicle screws pass through the isthmus of pedicle.

(3) D_AVC−PGVs_: the length between the anterior vertebral cortex (AVC) and the PGVs with different INTAs.

(4) D_O−AVC_: the distance between the O and the AVC with different INTAs.

(5) D_O−PGVs_: the distance between the O and the PGVs with different INTAs.

(6) In this study, abdominal aortic injury was mainly considered at L1-L3, arterial and venous injury was considered at L1-S1 (Fig. 1). With different INTAs, the D_AVC−PGVs_ are divided into four grades: D_AVC−PGVs_ ≤ 3 mm was Grade III, 3 mm < D_AVC−PGVs_ ≤ 5 mm was Grade II, D_AVC−PGVs_ > 5 mm was Grade I, and the untouching PGVs was recorded as N. Percentage of each grade = grade number/total number×100%.

### Statistical analysis

The statistical analysis was conducted using a standard SPSS 26.0 (SPSS Institute, Chicago, IL) software package. Data were expressed as the mean *±* standard deviation. A two-tailed Fisher’s exact test was performed to analyze the differences of the potential risk of PGVs between the left and right sides. The average D_AVC−PGVs_ and D_O−PGVs_ differences between the left and right sides were analyzed by T test, respectively. The D_O−AVC_ differences among different INTAs were analyzed by one-way analysis of variance (ANOVA). P values (< 0.05) were considered statistically significant.

## Results

**Fig. 3:**
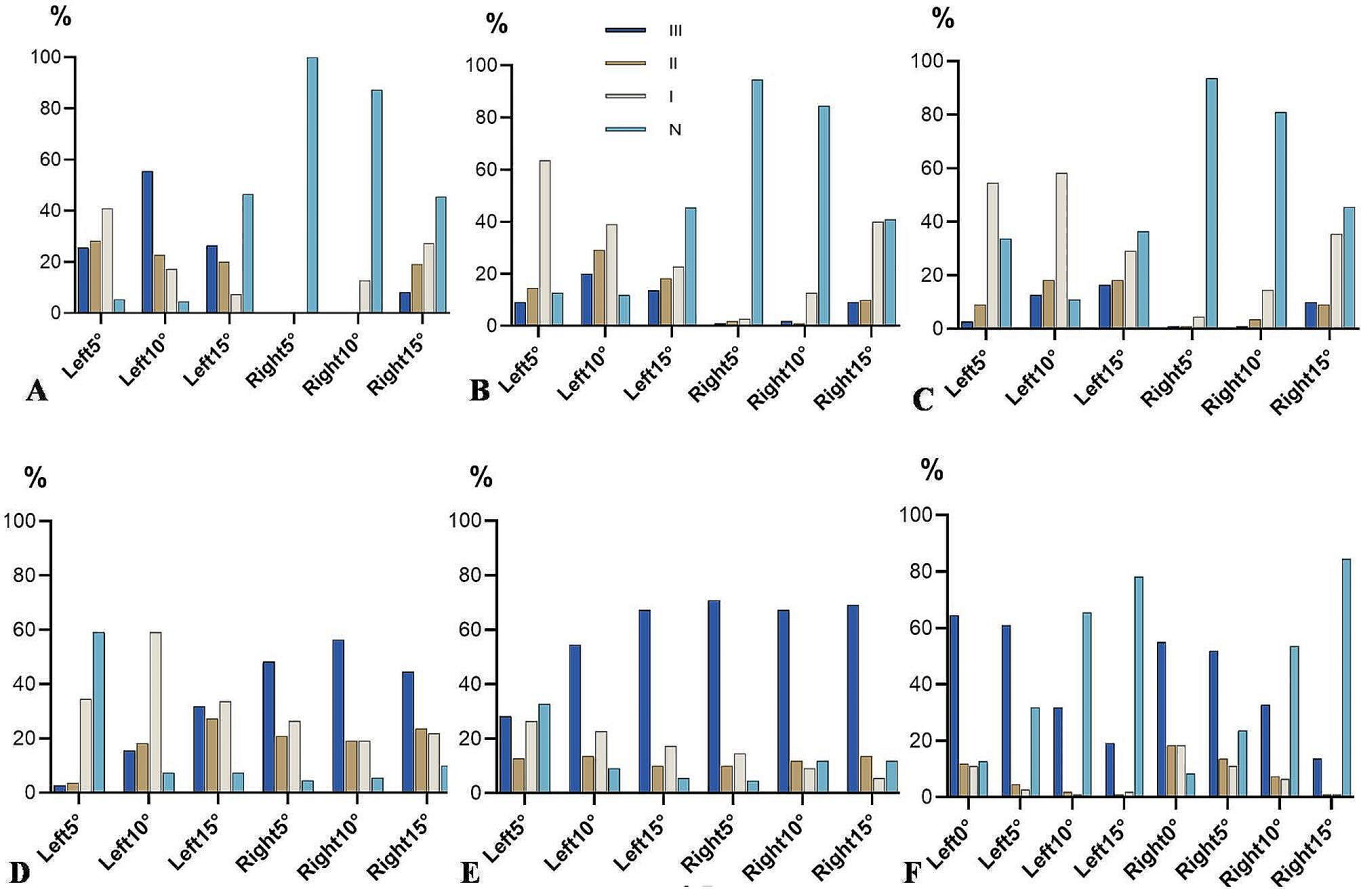
Ratio of potential damage risk to anterior vertebral vessels caused by left and right pedicle screws with different INTA (L1: figure **A**, L2: figure **B**, L3: figure **C**, L4: figure **D**, L5: figure **E**, S1: figure **F**). Grade III, II, I and N represent respectively: D_AVC−PGVs_ ≤ 3 mm, 3 mm < D_AVC−PGVs_ ≤ 5 mm, D_AVC−PGVs_ > 5 mm, and no touching of blood vessels. D_AVC−PGV_: the distance between AVC and PGV, AVC: anterior vertebral cortex, PGV: prevertebral great vessels, INTA: introversion angles, L: lumbar, S: sacral

A total of 110 consecutive patients with LDH (mean age: 61.43 ± 7.56 yrs) were included in the present study. No significant difference of age between male and female was found (*P* > 0.05). According to the Grade III, Grade II, Grade I, and N, the potential risk of PGVs at L1-S1 were shown in Table 1; Fig. 3. At L1-L2 with INTA5°, 10°, 15°, there were significant differences between the left and right sides (*P* < 0.01). Left side of L1, the incidence of Grade III was lowest at 5° (25.5%), and the highest was at 10° (55.5%). Right side of L1, N has the highest incidence at 5° (100%). Left side of L2, the Grade III was lowest at 5° (9.1%), and the highest was at 10° (20.0%). Right side of L2, the Grade III was lowest at 5° (0.9%), and the highest was at 15° (9.1%).At the L3-L4 with INTA 5°, 10°, there were significant differences between left and right side(*P* < 0.01), but the constituent ratios were not significantly different at 15° (*P* > 0.05). At the L5 with INTA 5°, 10°, 15°, obvious differences between left and right were found(*P* < 0.01). At the S1 with INTA 5°, 10°, there were significant differences between left and right, but the constituent ratios were not significantly different at 0°, 15° (*P* > 0.05).

**Table 1:** The type of DAVC−PGV with different INTA and the difference of DAVC−PGV (mm) between left side and right side at L1-S1

Lumbar	ITNA (°)	Type of D_AVC−PGV_	Left number (%)	Right number (%)	*P*	Left L_AVC−PGV_	Right L_AVC−PGV_
L1	5	Grade III Grade II Grade I N	28(25.5) 31(28.2) 45(40.9) 6(5.4)	- - - 110(100)	0.000	2.01 ± 0.65 3.94 ± 0.56 7.17 ± 1.79 -	- - - -
L1	10	Grade III Grade II Grade II N	61(55.5) 25(22.7) 19(17.3) 5(4.5)	- - 14(12.7) 96(87.3)	0.000	1.77 ± 0.67 3.66 ± 0.53 7.22 ± 1.83 -	8.56 ± 2.45 -
L1	15	Grade III Grade II Grade I N	29(26.4) 22(20.0) 8(7.3) 51(46.4)	9(8.2) 21(19.1) 30(27.3) 50(45.5)	0.000	1.77 ± 0.72 4.12 ± 0.67 7.12 ± 1.44-	2.35 ± 0.38 3.86 ± 0.55 7.74 ± 2.26 -
L2	5	Grade III Grade II Grade I N	10(9.1) 16(14.5) 70(63.6) 14(12.7)	1(0.9) 2(1.8) 3(2.7) 104(94.5)	0.000	2.13 ± 0.58 3.92 ± 0.58 8.56 ± 2.93 -	2.20 4.85 ± 0.19 10.58 ± 3.95 -
L2	10	Grade III Grade II Grade I N	22(20.0) 32(29.1) 43(39.1) 13(11.8)	2(1.8) 1(0.9) 14(12.7) 93(84.5)	0.000	1.96 ± 0.72 3.91 ± 0.63 7.87 ± 2.54 -	2.25 ± 0.06 3.27 8.87 ± 3.45 -
L2	15	Grade III Grade II Grade I N	15(13.6) 20(18.2) 25(22.7) 50(45.5)	10(9.1) 11(10.0) 44(40.0) 45(40.9)	0.027	2.05 ± 0.52 3.71 ± 0.64 7.20 ± 2.52 -	2.36 ± 0.67 4.27 ± 0.44 7.81 ± 2.42 -
L3	5	Grade III Grade II Grade I N	3(2.7) 10(9.1) 60(54.5) 37(33.7)	1(0.9) 1(0.9) 5(4.5) 103(93.6)	0.000	2.20 ± 0.11 4.05 ± 0.50 9.65 ± 2.94 -	1.47 3.94 6.53 ± 1.37 -
L3	10	Grade III Grade II Grade I N	14(12.7) 20(18.2) 64(58.2) 12(10.9)	1(0.9) 4(3.6) 16(14.5) 89(80.9)	0.000	2.08 ± 0.55 4.32 ± 0.53 8.44 ± 2.65 -	1.05 3.77 ± 0.73 7.91 ± 2.88 -
L3	15	Grade III Grade II Grade I N	18(16.4) 20(18.2) 32(29.1) 40(36.4)	11(10.0) 10(9.1) 39(35.5) 50(45.5)	0.079	2.06 ± 0.60 3.97 ± 0.50 7.73 ± 1.85 -	2.27 ± 0.57 4.30 ± 0.49 8.74 ± 2.36
L4	5	Grade III Grade II Grade I N	3(2.7) 4(3.6) 38(34.5) 65(59.1)	53(48.2) 23(20.9) 29(26.4) 5(4.5)	0.000	1.57 ± 0.99 3.89 ± 0.88 9.52 ± 3.57 -	1.76 ± 0.72 3.81 ± 0.50 7.49 ± 2.42 -
L4	10	Grade III Grade II Grade I N	17(15.5) 20(18.2) 65(59.1) 8(7.3)	62(56.4) 21(19.1) 21(19.1) 6(5.5)	0.000	1.86 ± 0.81 4.02 ± 0.55 8.13 ± 2.77-	1.58 ± 0.69 3.73 ± 0.50 7.66 ± 2.69 -
L4	15	Grade III Grade II Grade I N	35(31.8) 30(27.3) 37(33.6) 8(7.3)	49(44.5) 26(23.6) 24(21.8) 11(10.0)	0.118	1.78 ± 0.71 3.96 ± 0.51 8.20 ± 2.66 -	1.55 ± 0.64 4.13 ± 0.55 8.38 ± 2.47 -
L5	5	Grade III Grade II Grade I N	31(28.2) 14(12.7) 29(26.4) 36(32.7)	78(70.9) 11(10.0) 16(14.5) 5(4.5)	0.000	1.53 ± 0.70 3.87 ± 0.63 8.37 ± 3.63 -	1.30 ± 0.55 3.91 ± 0.56 7.64 ± 2.04 -
L5	10	Grade III Grade II Grade I N	60(54.5) 15(13.6) 25(22.7) 10(9.1)	74(67.3) 13(11.8) 10(9.1) 13(11.8)	0.037	1.43 ± 0.62 4.14 ± 0.69 8.42 ± 3.03 -	1.29 ± 0.56 3.90 ± 0.55 6.99 ± 1.57 -
L5	15	Grade III Grade II Grade I N	74(67.3) 11(10.0) 19(17.3) 6(5.5)	76(69.1) 15(13.6) 6(5.5) 13(11.8)	0.019	1.20 ± 0.44 3.92 ± 0.66 8.94 ± 3.37 -	1.27 ± 0.44 4.04 ± 0.51 7.77 ± 1.95 -
S1	0	Grade III Grade II Grade I N	71(64.5) 13(11.8) 12(10.9) 14(12.7)	60(55.0) 20(18.3) 20(18.3) 9(8.3)	0.142	1.26 ± 0.51 3.77 ± 0.70 6.69 ± 1.63 -	1.48 ± 0.61 3.99 ± 060 7.91 ± 3.04 -
S1	5	Grade III Grade II Grade I N	67(60.9) 5(4.5) 3(2.7) 35(31.8)	57(51.8) 15(13.6) 12(10.9) 26(23.6)	0.005	1.17 ± 0.44 3.96 ± 0.90 5.59 ± 0.58 -	1.39 ± 0.57 3.77 ± 0.70 8.34 ± 2.87 -
S1	10	Grade III Grade II Grade I N	35(31.8) 2(1.8) 1(0.9) 72(65.5)	36(32.7) 8(7.3) 7(6.4) 59(53.6)	0.024	1.15 ± 0.46 3.74 ± 0.57 5.13 -	1.41 ± 0.60 4.00 ± 0.41 6.54 ± 2.20 -
S1	15	Grade III Grade II Grade I N	21(19.1) 1(0.9) 2(1.8) 86(78.2)	15(13.6) 1(0.9) 1(0.9) 93(84.5)	0.727	1.08 ± 0.35 3.70 7.78 ± 0.55	1.33 ± 0.54 4.31 7.83

D_AVC−PGV_: the distance between AVC and PGV, AVC: anterior vertebral cortex, PGV: prevertebral great vessels, INTA: introversion angles, L: lumbar, S: sacral, SD: standard deviation. Grade III: D_AVC−PGVs_ ≤ 3 mm, Grade II: 3 mm < D_AVC−PGVs_ ≤ 5 mm, Grade I: D_AVC−PGVs_ > 5 mm, N: non-contact

**Fig. 4:**
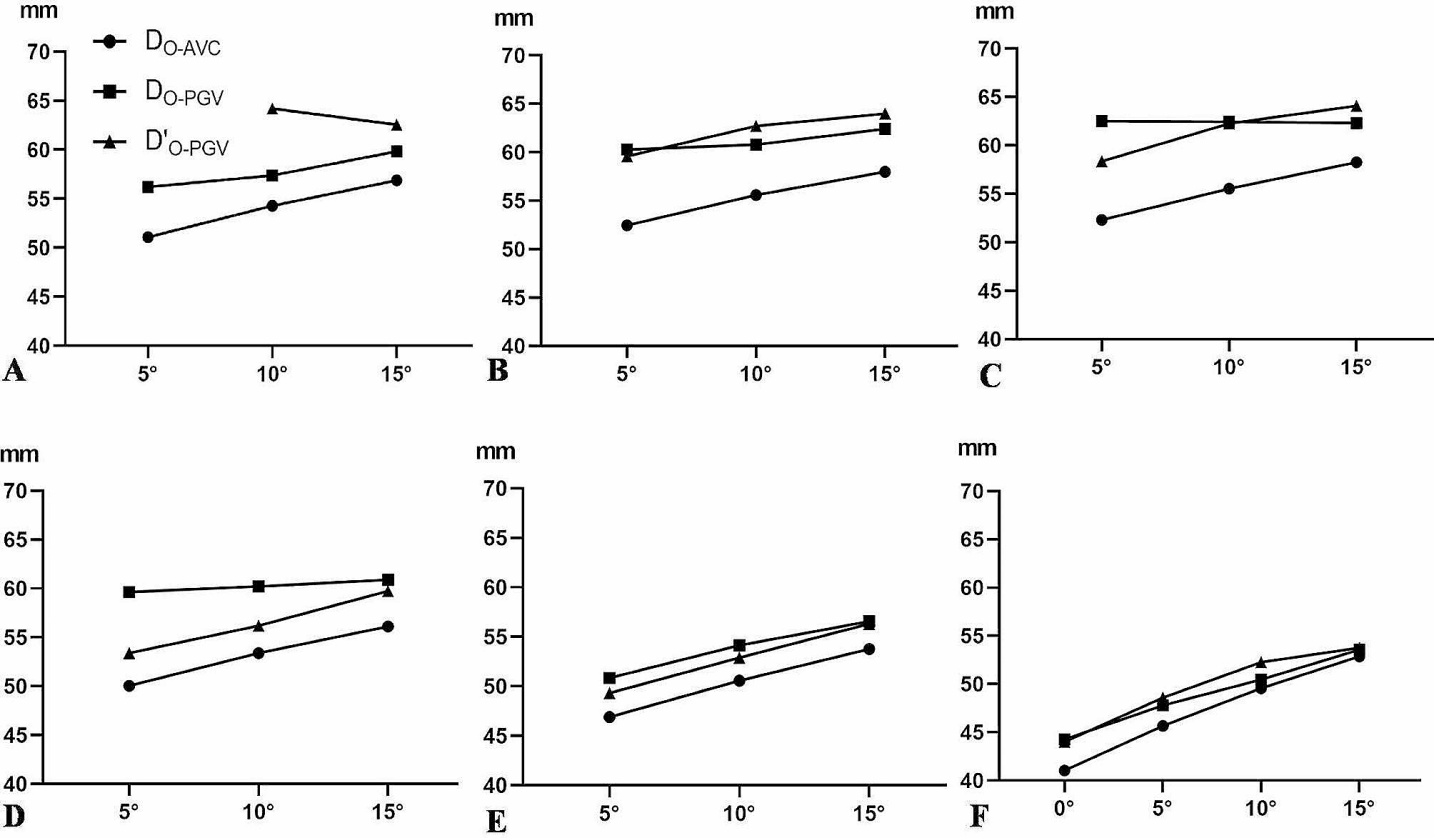
The maximum length of pedicle screw insertion (D_O−AVC_) and the distance between the entry point of the left pedicle screw and the anterior vertebral vessels (D_O−PGVs_). D’_O−PGVs_ represent the distance between the entry point of the right pedicle screw and the anterior vertebral artery. Figure **A**-**E** were The length variation diagrams of D_O−AVC_, D_O−PGVs_ and D’_O−PGVs_ with INTA 5°, 10° and 15° at L1-L5, respectively; **F** was the length variation diagram of D_O−AVC_, D_O−PGVs_ and D’_O−PGVs_ with INTA 0°, 5°, 10° and 15° at S1. AVC: anterior vertebral cortex, PGV: prevertebral great vessels, INTA: introversion angles, L: lumbar, S: sacral

The D_O-AVCs_ and D_O-PGVs_ were shown in Table 2; Fig. 4. The D_O-AVCs_ were significant differences at different INTAs of L1-S1 (*P* < 0.01). When INTA was 5°, the maximum distance was measured at L2 and L3 (52.47 mm, 52.31 mm), the minimum distance was measured at S1 (41.03 mm). At L1-L4, the INTA increased 5°, the screw length increased 3 mm. At L5-S1, the INTA increased 5°, the screw length increased 4 mm. At L1 D_O-PGVs_, there were significant difference between the left side and right side (*P* < 0.01), and the minimum distance of left was 56.21 mm. At L2, there were no marked difference of D_O-PGVs_ between left side and right side (*P* > 0.05), and when INTA was 5°, the average distance was 60 mm (range, 48–73 mm). At L3, when INTA was 5° and 10°, there were no significant difference of D_O-PGVs_ between the left and right side (*P* > 0.05), the distance of left side was 62.50 mm, the right side was 58.37 mm. However, the left and right of D_O-PGVs_ were significant difference when INTA was 15° (*P* < 0.05). For L4 D_O-PGVs_, there were significant differences between the left and right side when INTA was 5° and 10° (*P* < 0.05), but no significant difference at 15° (*P* > 0.05).No significant difference of L5 D_O-PGVs_ between the left and right side was found (*P* > 0.05), the distance of left was 50.84 mm when INTA was 5°, and right side was 56.31 mm when INTA was 10°. As for S1 D_O-PGVs_, there were no marked difference between the left and right side (*P* > 0.05), the distance of left side and right side was 44.26 mm, 44.01 mm when INTA was 5°, respectively; and 53.57 mm, 53.76 mm when INTA was 15°, respectively.

**Table 2:** The average D_O−AVC_ (mm) with different INTAs and the difference of D_O−PGV_ (mm) between left side and right side at L1-S1

Lumbar	INTA (°)	D_O−AVC_ (mean + SD)	*P* (D_O−AVC_)	Left D_O−PGV_ (mean *±* SD)	Right D_O−PGV_ (mean *±* SD)	*P* (D_O−PGV_)
L1	5 10 15	51.09 ± 4.30 54.30 ± 4.16 56.88 ± 4.17	0.000	56.21 ± 5.31 57.40 ± 4.80 59.83 ± 4.99	64.21 ± 4.38 62.57 ± 4.80	0.000 0.002
L2	5 10 15	52.47 ± 4.17 55.58 ± 4.01 57.99 ± 3.95	0.000	60.29 ± 5.47 60.78 ± 5.27 62.41 ± 4.62	59.58 ± 4.25 62.72 ± 6.59 63.99 ± 5.25	0.755 0.180 0.070
L3	5 10 15	52.31 ± 5.41 55.55 ± 4.96 58.26 ± 4.87	0.000	62.50 ± 6.36 62.43 ± 5.86 62.32 ± 6.02	58.37 ± 5.83 62.26 ± 6.68 64.08 ± 6.43	0.103 0.910 0.025
L4	5 10 15	50.03 ± 5.41 53.39 ± 5.26 56.11 ± 5.29	0.000	59.64 ± 6.77 60.20 ± 6.11 60.90 ± 5.99	53.38 ± 5.88 56.18 ± 6.65 59.73 ± 7.18	0.000 0.000 0.107
L5	5 10 15	46.90 ± 6.08 50.57 ± 5.90 53.77 ± 5.59	0.000	50.84 ± 6.85 54.15 ± 7.22 56.56 ± 6.73	49.32 ± 6.06 52.90 ± 5.97 56.31 ± 5.56	0.120 0.186 0.776
S1	0 5 10 15	41.03 ± 5.72 45.67 ± 5.70 49.56 ± 5.47 52.87 ± 5.69	0.000	44.26 ± 6.03 47.80 ± 5.93 50.47 ± 5.25 53.57 ± 6.14	44.01 ± 5.12 48.59 ± 5.41 52.25 ± 4.74 53.76 ± 4.29	0.756 0.386 0.099 0.914

D_O−AVC_: the distance between the O and the AVC, D_O−PGV_: the distance between the O and the PGV, AVC: anterior vertebral cortex, PGV: prevertebral great vessels, INTA: introversion angles, L: lumbar, S: sacral, SD: standard deviation

The average D_AVC−PGVs_ with different INTAs of L1-S1 were shown in Table 3. At L1-L4, there were significant differences of D_AVC−PGVs_ between the left side and right side when INTA was 5, 10, 15° (*P* < 0.01) except 10° at L3. As for L5 D_AVC−PGVs_, notable differences of between left and right sides were found when INTA was both 5° and 10° (*P* < 0.01), but no significant difference when INTA was 15° (*P* > 0.05). At S1, there were obvious differences of D_AVC−PGVs_ between the left side and right side when INTA was 0°, 5° and 10° (*P* < 0.01), however, no significant difference was occurrence when INTA was 15° (*P* > 0.05).

**Table 3:** The average D_AVC−PGV_ (mm) with different INTA at L1-S1

Lumbar	INTA (°)	Left D_AVC−PGV_ (mean *±* SD)	Right D_AVC−PGV_ (mean *±* SD)	*P*
L1	5 10 15	4.93 ± 2.78 3.20 ± 2.26 3.38 ± 2.03	- 8.56 ± 2.45 5.57 ± 2.77	- 0.000 0.000
L2	5 10 15	7.07 ± 3.51 5.20 ± 2.85 4.54 ± 2.36	7.12 ± 4.61 8.33 ± 3.78 6.12 ± 2.72	0.003 0.000 0.000
L3	5 10 15	8.69 ± 4.19 6.69 ± 3.30 5.20 ± 2.77	5.53 ± 2.55 6.79 ± 3.28 6.81 ± 3.33	0.005 0.893 0.000
L4	5 10 15	8.49 ± 4.11 6.28 ± 3.40 4.75 ± 3.22	3.79 ± 2.80 3.24 ± 2.72 3.88 ± 3.07	0.000 0.000 0.014
L5	5 10 15	4.65 ± 3.88 3.58 ± 3.36 2.90 ± 3.33	2.54 ± 2.49 2.23 ± 1.98 2.10 ± 1.88	0.000 0.001 0.770
S1	0 5 10 15	2.28 ± 2.02 1.54 ± 1.19 1.39 ± 0.97 1.75 ± 7.97	3.26 ± 2.90 2.81 ± 2.72 2.52 ± 2.09 1.88 ± 1.77	0.002 0.000 0.000 0.155

D_AVC−PGV_: the distance between AVC and PGV, AVC: anterior vertebral cortex, PGV: prevertebral great vessels, INTA: introversion angles, L: lumbar, S: sacral, SD: standard deviation

## Discussion

Vascular injury during spine surgery is a devastating complication, the incidence depends on the anatomical region, surgical approach, and surgical technique [2, 3, 7, 11, 12]. Thus, the safety and accuracy of pedicle screws are of great concern to surgeons. Some studies pointed out that the robot-assisted system can not only visualize the probing hole but also the position of screw in real time, which could improve the safety of the operation [13, 14]. Also, some authors found the robot-assisted system can reduce the perforation rate [13, 15]. However, a meta-analysis including 15 eligible RCTs concluded no significant difference among the Orthbot-assisted technique, the Renaissance-assisted technique, the conventional freehand technique, and the Spine Assist-assisted technique in accuracy of pedicle screws was found [16]. Also, a systematic review and meta-analysis showed there was no significant difference of accuracy of pedicle screws with Robotic surgery and freehand/conventional surgery [7]. Therefore, there is no consensus on whether the new navigation system can improve the safety performance of pedicle screws. In addition, many medical centers do not have computer-assisted equipment and those are not widely used.

At present, the range of D_AVC−PGVs_ and D_O−PGVs_ with different INTAs in thoracic spine have been analyzed in some studies [8, 17, 18]. However, the relationship of D_O−PGVs_ and different INTAs at lumbosacral region has not been quantified at length. A study including a total of 9179 pedicle screws in the thoracic or lumbosacral spine found 210 (2.3%) malpositioned screws with freehand pedicle screw placement, eleven screws (0.12%) were significantly malpositioned and required a second operation for screw revision [19]. Foxx et al. retrospectively analyzed 680 pedicle screws distributing thoracolumbar and lumbosacral fusion, and found 33 of those were in contact with the great vessels, including 4 cases of the aorta, 7 cases of the iliac artery, and 22 cases of the iliac vein. No patients developed any symptoms or sequelae due to contact between the great vessels and pedicle screws during the 44-month follow-up period [20]. Early study has shown that screw misplacement was 6.5%, Screw breakage occurred in 12.4% of the patients [2]. With the popularization and proficiency of pedicle screw technology, the accuracy of screw insertion is also constantly improving. Based on anatomical features, if the screw penetrated unfortunately cortex of vertebrae with the tip to the appropriate length, the structures in front of the vertebral body such as large blood vessels, would not be in danger. The intraoperative INTA is closely related to the length of pedicle screws and the rupture of pedicle wall. Excessive INTA of screws can easily enter the spinal canal and damage nerves; and the INTA is too small, the screw may be located outside the pedicle, resulting in insufficient screw holding force and injury of blood vessels and nerves. From the present results, the recommended INTA was 5° at L1-L2, left 0–10° and right 0–15° at L3, left was 10° and right 0–5° at L4, left 15° and right 0–5° at L5, 0–15° at S1.

Studies found bicortical pedicle screw especially for patients with osteoporosis, can increase in depth of insertion of the pedicle screw resulting in higher pullout force and energy, and the stress was dispersed between the two cortical bone, so that the fixation strength of cortical bone was significantly higher than the cancellous bone [18, 21]. Some authors suggested the bicortical pedicle screw should penetrate with the tip no more than 1 thread beyond the cortical surface [22], and another authors proposed that the screw tip should penetrate no more than 2 mm through the anterior cortex of the vertebral body [23]. Some studies pointed out the perforation range was about 2–4 mm, so the vascular that the distance of D_AVC−PGVs_ less than 3 mm are great injured risk [24, 25]. Therefore, considering the PGVs might be irritated due to their pulsating, the appropriate safe distance between the bicortical pedicle screw and the PGV was approximately 5 mm. Under normal conditions, with mastering screw insertion technology and the help of X-ray fluoroscopy assistance, it was unlikely for the bicortical pedicle screw to exceed 5 mm in front of the vertebral body. Therefore, when the D_AVC−PGV_ was greater than 5 mm, the bicortical pedicle screw was not likely to damage the PGVs. However, if the D_AVC−PGV_ was less than 5 mm, the bicortical pedicle screw had a higher risk of injuring the PGVs due to the narrow a vascular space in front of the vertebral body. The smaller the D_AVC−PGV_ is, the higher the risk is to injury the PGV. In the present study, when INTA was 5°, the risk of vertebral vascular injury was greater than 10% except for the right side of L1, the left and right sides of L2-L3, and the left side of L4, this finding similar to other studies [8, 9]. In addition, our results found at L5-S1, the risk of vascular injury was highest, the average distance of D_AVC−PGVs_ less than 4 mm, indicating that once the pedicle screw penetrates the prevertebral cortex, the prevertebral blood vessels will inevitably be damaged. At L5-S1, the present results showed the D_O−AVC_ was 40 mm to 50 mm, meaning that our choice of screw length is limited. Due to the limitation of anatomical structure at S1, the theoretical optimal INTAs may not be realized, only 35 mm pedicle screw can be selected when INTA was 0°, and 40 mm pedicle screw can be selected when INTA was 5 °, but shorter screw depth could not achieve adequate stability in patients with osteoporosis. The present result also indicated when the INTAs increased by 5°, the screw depth increased by 3 mm to 4 mm, therefore, we recommend using 3 mm increments in screw length to reduce vascular damage and increase stability.

Major vascular injury is a known complication of spinal surgery. according to our study result, the possibility that pedicle screw contacted with arteries was more than the possibility contacted with veins at L4, right pedicle screw was more likely to contact with veins than arteries at L5, left pedicle screw was more likely to contact with veins (e.g. left common iliac vein) than arteries, right pedicle screw was more likely to contact with arteries (e.g. right internal iliac artery) than veins (e.g. right common iliac vein) at S1. In summary, we should focus on arterial damage at L4, vein damage at the right of L5 and left common iliac vein and right internal iliac artery at S1.

Our study has certain limitations. First, the data have unavoidable measurement errors. Second, all CT images were taken in the supine position, and the traditional surgical position is the prone position. However, Riccio et al [26] found that the inferior vena cava and the abdominal aorta in the lumbar region is steady whatever in the prone and supine positions. On the other hand, Li Zhao et al [18] found that lumbar lordosis did not significantly affect the distance between the lumbar spine and PGVs at any level. Finally, because screw implantation is not only at the axial plane of the vertebral body but also has a difference in screw length depending on the head and tail inclination in the sagittal plane, further study should be carried out to assess the situation.

## Conclusions

According to the analyzed of different parameters, L1-S1 vascular damage potential risks were different; the risk of L5-S1 was the highest. Therefore, the screw length was limited and the anterior vertebral cortex should be avoided penetrated at L5-S1. Meanwhile, we recommend using 3 mm increments in screw length to reduce vascular damage. And left common iliac vein and the right internal iliac artery damage should be paid more attention when pedicle screw is placed at S1.

## Data Availability

All data generated or analyzed during this study are included in this published article.
